# AI-Based Classification of Multiple Sclerosis Using OCT Retinal Layer Thickness Across Two Centers

**DOI:** 10.3390/biomedicines14071613

**Published:** 2026-07-17

**Authors:** Miguel Ortiz, Javier Dongil-Moreno, Gema Rebolleda, Naiara Artiaga, Luciano Boquete, Juan M. Miguel-Jimenez, Maria J. Rodrigo, Almudena López-Dorado, Rosario Zamora, Eduardo García Vicente, Eva M. Sánchez-Morla, Beatriz Andres-Luna, Francisco J. Muñoz Negrete, Elena Garcia-Martin

**Affiliations:** 1 School of Physics, University of Melbourne, Melbourne, VIC 3010, Australia; 2 Biomedical Engineering Group, Department of Electronics, University of Alcalá, 28801 Alcalá de Henares, Spainjmanuel.miguel@uah.es (J.M.M.-J.); almudena.lopez@uah.es (A.L.-D.); 3 Ophthalmology Service, Ramon y Cajal University Hospital, IRYCIS, 28034 Madrid, Spain; 4 Department of Surgery, Medical and Social Sciences, University of Alcalá, 28801 Alcalá de Henares, Spain; 5 Department of Ophthalmology, Miguel Servet University Hospital, 50009 Zaragoza, Spainmariajesusrodrigo@hotmail.es (M.J.R.); 6 Miguel Servet Ophthalmology Research Group (GIMSO), Aragon Health Research Institute (IIS Aragon), Biotech Vision Center, Quirón Ophthalmology Institute, University of Zaragoza, 50009 Zaragoza, Spain; 7 Institute of Psychiatry and Mental Health, Gregorio Marañón University General Hospital, IiSGM, 28007 Madrid, Spain; emsmorla@gmail.com; 8 Faculty of Medicine, Complutense University of Madrid, 28040 Madrid, Spain

**Keywords:** multiple sclerosis, optic nerve, optical coherence tomography, artificial intelligence, support vector machine classifier, Shapley additive explanations

## Abstract

**Background:** The latest revision of the McDonald criteria for diagnosis of multiple sclerosis (MS) establishes that the optic nerve can serve as a fifth anatomical location within the central nervous system for diagnosis. Optical coherence tomography (OCT) images can serve as evidence for this purpose. **Objective:** To assess the accuracy of automated artificial-intelligence-based classification of MS patients using OCT data obtained from two different centers. **Methods:** OCT data were collected from two centers using standardized APOSTEL-based protocols and similar equipment. Retinal layer thicknesses—mean and standard deviation (STD) values—were analyzed in four layers and in six regions per layer per eye. A support vector machine classifier with recursive feature elimination and Shapley additive explanations value analysis was applied to identify the most relevant features and maximize classification accuracy between control subject and MS patient eyes. **Results:** The database drawn from two hospitals comprised 112 eyes with MS without prior history of optic neuritis and 193 eyes of control subjects. The classifier achieved maximum accuracy (0.8459) using 20 input features. The mean and STD metrics had similar importance, with the most influential layers being the ganglion cell layer, inner plexiform layer, and the inner retinal layer complex. Key regions included the papillomacular bundle and the superior temporal perimacular area. **Conclusions:** OCT data facilitates highly accurate MS diagnosis across different centers. Artificial intelligence assessment could facilitate automated classification. These findings provide evidence of the important role of the optic nerve in MS diagnosis.

## 1. Introduction

Multiple sclerosis (MS) diagnosis is based on the McDonald criteria, which require evidence of dissemination in space (DIS) and time (DIT) of demyelinating lesions in the central nervous system (CNS). These disseminations are detected by clinical evaluation, imaging, and laboratory tests.

The recent 2024 revisions of the McDonald criteria establish that the optic nerve—assessed by MRI, visual evoked potentials, or optical coherence tomography (OCT)—can serve as a fifth anatomical location within the CNS for diagnosis, if no better explanation exists for optic nerve pathology [1].

Improvements in MS diagnostic criteria have increased sensitivity and specificity, thereby reducing the length of time between disease onset and definitive diagnosis [2]. Earlier diagnosis and consequent treatment of MS patients positively influence long-term outcomes [3,4]. It is recommended that the timeline between referral and completion of diagnostic investigation in suspected MS cases should not exceed 12 [5] or even 7 weeks [6]. However, diagnostic delays in MS are common, often due to early misdiagnosis, and are associated with greater disease burden [7], older age at onset, lower education level, and motor symptoms at onset [8].

The frequency of misdiagnosis (incorrect assignment of an MS diagnosis) ranges from 5% to 41% and that of underdiagnosis (unrecognized MS) ranges from 3% to 58% [9]. Misdiagnosis can lead to unnecessary exposure to immunosuppressive or immunomodulatory disease-modifying therapies.

OCT allows clinicians to quantify retinal layer atrophy due to neurodegeneration in MS. It is non-invasive, well tolerated by patients, performed quickly, uses relatively affordable equipment, and follows widely accepted protocols [10].

Several studies have demonstrated structural retinal alterations in MS patients both with [11,12] and without prior episodes of optic neuritis (ON). The prior literature also describes artificial-intelligence-based MS diagnosis decision support systems [13,14,15,16], with some publications that recommend the use of OCT as an additional tool to support MS diagnosis [17,18,19,20,21].

Validation of any automated diagnostic tool requires multicenter data to ensure reproducibility and comparability. Few studies to date have used multicenter OCT data for MS diagnosis [21].

The aim of this study—conducted across two hospitals—was to evaluate the effectiveness of an MS diagnosis system based on artificial intelligence (AI) in a multicenter framework. To this end, we ensured full comparability of OCT devices (same model, acquisition protocols, and review and calibration procedures) and analyzed the combined data.

## 2. Material and Methods

Patients with MS diagnosis based on the McDonald criteria were included in the study. All study participants were Caucasian. Eyes with a clinical history of optic neuritis were excluded because the aim was to find a diagnostic method for cases that are difficult to detect or for early detection. When a patient had one eye with a history of optic neuritis and the other without, only the eye without optic neuritis was included.

The exclusion criteria were clinical history of optic neuritis in this eye, axial length longer than 25.2 mm, refractive errors ≥5 diopters (D) of equivalent spherical or ≥3 D of astigmatism. The eyes had no concomitant ocular diseases, nor any previous history of retinal pathology, glaucoma, amblyopia, or systemic conditions that could affect the visual system. In order to expand the database, in most cases both subject eyes were included; therefore, the principle of independence was not strictly adhered to in the statistical tests performed.

The study was approved by the local ethics committees (C.I. PI21/113) and was conducted in accordance with the Declaration of Helsinki. Both protocols include provisions for the use of data in multicenter studies. Written informed consent was obtained from all participants prior to enrollment.

Visual acuity was measured in all eyes using the Snellen chart. The anterior segment, including the lens, was examined and intraocular pressure was measured using applanation. The fundus was also examined using OCT. In this way, it was confirmed that the eyes met the criteria and did not have any other conditions that would prevent participation in the study.

### 2.1. OCT Acquisition

The OCT data acquisition procedures at both centers adhered to the APOSTEL recommendations [10], employing similar OCT devices, standardized protocols, and identical calibration processes.

OCT imaging was performed by experienced neuro-ophthalmologists at both hospitals using a Heidelberg Engineering Spectralis spectral-domain device (Heidelberg Engineering, Heidelberg, Germany). The system employed automatic real-time averaging, active eye tracking, and an anatomical positioning system in a dark room environment without pupil dilation. Data were acquired using a macular volume scan with eye tracking, covering a 25° × 30° field, comprising 61 vertical B-scans with 768 A-scans per B-scan. The posterior pole asymmetry analysis protocol used represented the retinal thickness on an 8 × 8 grid [22].

### 2.2. Retinal Thickness Evaluation

Retinal layer segmentation was automated using the proprietary software algorithm (HEYEX v2.5.4, HRA version 6.9.a) and was manually corrected when necessary. All scans underwent quality control based on the latest OSCAR-IB criteria [23] to ensure sufficient image quality.

**Figure 1:**
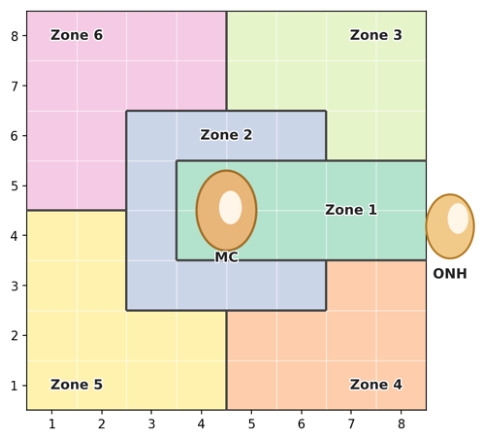
Representation of the areas analyzed, with ONH being the position of the optic nerve head, MC being the position of the macula, and the 6 defined areas being Zone 1 (papillomacular bundle that connects the area of maximum vision of the eye—the macula—to the optic nerve exit), Zone 2 (paramacular), Zone 3 (upper nasal perimacular), Zone 4 (lower nasal perimacular), Zone 5 (lower temporal perimacular), and Zone 6 (upper temporal perimacular). Abbreviations: ONH: optical nerve head. MC: macula.

Consistent with previous studies [16,24] demonstrating that MS primarily affects the inner retinal layers, thickness measurements focused on the retinal nerve fiber layer (RNFL), ganglion cell layer (GCL), inner plexiform layer (IPL), and their combined structure, the inner retinal layer (IRL) complex (IRL: RNFL + GCL + IPL). These layers were analyzed within the anatomically defined regions illustrated in Figure 1.

The unit of analysis was the eye. Mean and standard deviation (STD) retinal layer thickness values were calculated for each of the 6 predefined anatomical zones and the 4 retinal structures, resulting in a total of 48 variables (6 zones × 2 metrics × 4 layers).

### 2.3. Statistical Analysis

Continuous variables were expressed as mean ± standard deviations (normal distribution) or median and range (nonnormal distribution), and categorical variables as numbers and percentages. Normality was assessed using the Shapiro–Wilk test for sample sizes < 50 and the Kolmogorov–Smirnov test for sample sizes of ≥ 50. Statistical significance for continuous variables was evaluated using the non-parametric Mann–Whitney U test, while Pearson’s chi-square test was applied to categorical variables. A *p*-value < 0.05 was considered statistically significant. OCT thickness differences between controls and MS were represented graphically using the area under the receiver operating characteristic curve (AUC).

### 2.4. AI Classification

OCT data from control subjects and people with multiple sclerosis (PwMS) from both hospitals were combined into a single dataset for AI classification.

Five classifier models coupled with recursive feature elimination (RFE) to enhance model reliability by removing less relevant variables and improving explainability were employed [25]. The RFE process optimized accuracy using leave-one-out (LOO) cross validation. In each iteration, feature importance was ranked using Shapley additive explanations (SHAP) [26] to calculate the mean absolute SHAP value for each feature; features with lower mean values were considered less important and subsequently removed.

The procedure was as follows:

1. Each classifier was trained using the initial set of 48 features (F_N_ = 48) and applying LOO cross validation. At each step, the model was trained on all eyes except one, which was used for validation, and SHAP values for each input feature were computed. After completing the LOO cycle, a confusion matrix and overall accuracy were calculated, along with mean SHAP values per feature.
2. The least important feature (lowest mean SHAP value) was eliminated, reducing the number of features by one (F_N_ = F_N_ − 1), and Step 1 was repeated until only one feature remained.

This RFE approach identified the feature subset that yielded the highest classification accuracy.

## 3. Results

Table 1 reports the main demographic and clinical characteristics of the PwMS and healthy controls. In the PwMS cohort, the number of patients in the MSH (Miguel Servet Hospital) group was higher than in the RCH (Ramón y Cajal Hospital) group, and there was a significant difference in both disease duration and Expanded Disability Status Scale (EDSS) score. In the control group, there was also an imbalance in the number of subjects, but no significant difference in the male-to-female ratio or the age of the participants.

As indicated, OCT data from both hospitals were combined into a single dataset for classification.

### 3.1. OCT Thickness Comparison

**Figure 2:**
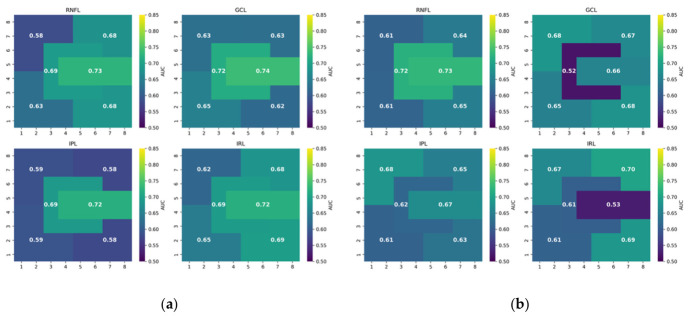
AUC values of thickness values. (**a**) Mean variable. (**b**) STD variable.

Figure 2 shows the AUC values of the mean and STD variables for controls and patients (MS).

For the mean variable, the highest AUC values in the four retinal structures analyzed are found in Zone 1, presenting values ≥ 0.72. The next most affected zone by AUC value is Zone 2, presenting values between 0.69 and 0.72.

For the STD variable, the greatest difference between controls and PwMS occurs in the RNFL (Zone 1 and Zone 2), followed by Zones 3 and 4 of the IRL complex.

Table 2 shows a detailed analysis of the AUC values.

### 3.2. Classification

To validate the choice of classifier, several machine learning methods were benchmarked using the same evaluation protocol: LOO cross-validation with RFE, applied to the combined dataset of 305 eyes from two hospitals. Five classifiers were compared: Support Vector Machine with RBF kernel (SVM-RBF), Support Vector Machine with linear kernel (SVM-Linear), Logistic Regression (LR), Linear Discriminant Analysis (LDA), and K-Nearest Neighbors (KNN). For each method, hyperparameters were tuned via grid search, and the feature subset yielding the highest overall accuracy was selected. As shown in Table 3**,** the SVM-RBF classifier achieved the best accuracy (84.59%) and the highest sensitivity (71.43%), confirming its suitability for this classification task. The hyperparameters of the SVM-RBF model, selected via grid search, are C = 5 and γ = 0.1.

**Figure 3:**
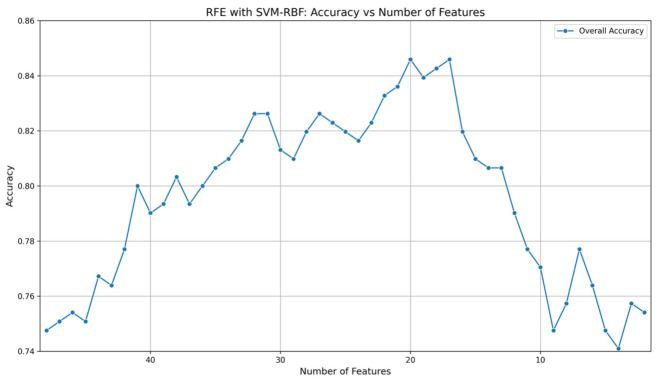
Representation of application of the RFE algorithm.

Figure 3 shows application of the RFE algorithm to the combined OCT dataset from both hospitals using an SVM as classifier. The graph illustrates how classification accuracy changes as the number of features decreases from the initial 48 down to 1.

**Figure 4:**
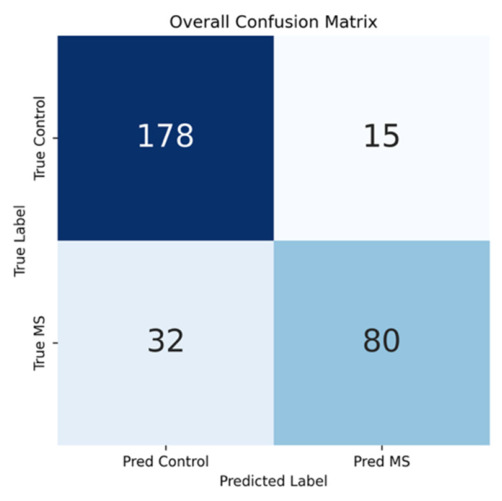
Confusion matrix obtained in the SVM-RBE classifier with the 20 best features selected by RFE.

The highest accuracy value (0.8459) is achieved when the classifier is fed with 20 or 17 features, resulting in the confusion matrix shown in Figure 4.

**Figure 5:**
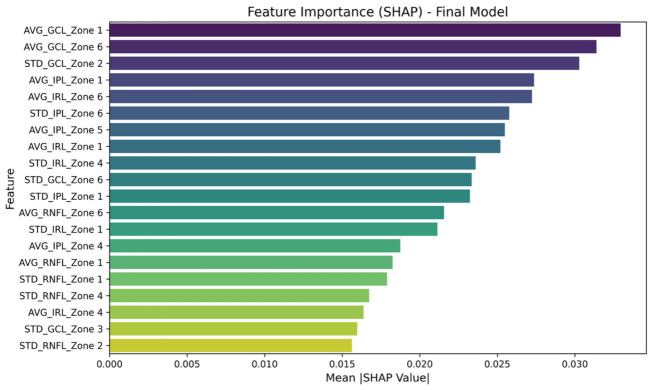
Ranking by SHAP value of the 20 characteristics that yield the best accuracy value.

Figure 5 displays the 20 features that yielded the highest classifier accuracy, ranked by SHAP importance. A cumulative analysis leads to three main conclusions:

- Metric importance: mean thickness contributes slightly more to model performance than standard deviation (weight 0.2448 vs. 0.2135).
- The most influential retinal layers, in order of importance, are the GCL (weight = 0.134), IPL (weight = 0.1207), IRL complex (weight = 0.1135), and RNFL (weight = 0.0901).
- Key anatomical regions: Zone 1 (weight = 0.1661) is the most discriminative, followed by Zone 6 (superotemporal quadrant, weight = 0.1293). The weights for the other zones are as follows: Zone 4 = 0.0755, Zone 2 = 0.0459, Zone 5 = 0.0255, and Zone 3 = 0.016.

**Figure 6:**
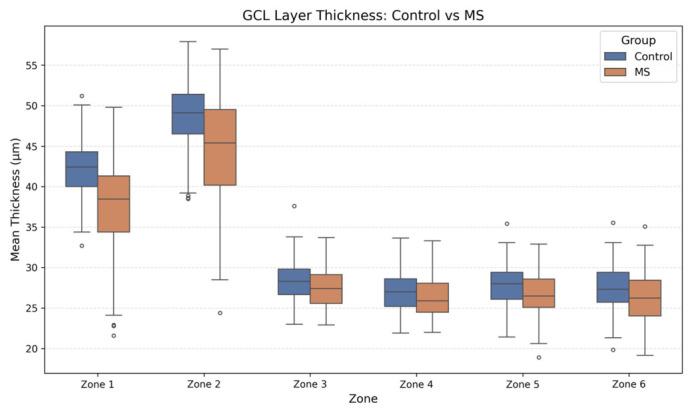
Representation of GCL thickness between Control and MS groups.

Since, according to the SHAP values, the GCL is the most influential in the classification, in Figure 6 the box plot is presented, which allows us to appreciate how, in MS patients, this structure is thinner than in the control subjects.

## 4. Discussion

A growing body of evidence supports the use of OCT as a reliable and non-invasive tool in MS diagnosis [18,21]. Previous studies have suggested that the retina itself may represent an early target of neurodegenerative and inflammatory processes in MS, leading to detectable neuronal damage from disease onset. In line with this, several authors have argued that OCT measurements may be particularly valuable for detecting neuroaxonal loss in early MS when conventional MRI brain atrophy metrics are less sensitive [27]. Based on the evidence, the revised 2024 McDonald criteria recognize the optic nerve as a fifth topographical location for dissemination in space [1].

In this multicenter study, we developed and validated an AI-based MS diagnosis support system using OCT data collected at two independent hospitals. Our approach achieved overall accuracy of 0.8459 while maintaining classifier decision interpretability through SHAP analysis. These findings highlight explainable AI’s potential to provide transparent diagnostic support in clinical practice.

Previous single-center studies have demonstrated that mean retinal thickness and inter-eye differences are particularly informative for detecting MS in patients without ON [16,24]. However, in our cohort, inter-eye difference could not be assessed because, in patients with unilateral ON, only the unaffected eye was included in the analysis. Interestingly, our results indicate that variability within regions, measured as the STD of retinal thickness, had similar importance to mean values (weights: 0.2448 vs. 0.2135). Although this observation requires confirmation in larger datasets, it may suggest that PwMS exhibit greater variability in retinal thickness compared with controls.

Using the mean and inter-eye difference variables, a single-center analysis [16,24] showed that the layers and regions most influencing the classifier were the GCL and Zones 2, 5, and 6. In this study, mean and STD variable analysis revealed that the layers with greatest impact on classifier performance were the GCL, IPL, and IRL, all with very similar influence, whereas the RNFL contributed less. Among these three most relevant structures, the primary regions of interest for the classifier were Zone 1 (papillomacular bundle) and Zone 6 (upper temporal perimacular).

Our findings are generally consistent with other studies. For example, [28] reported atrophy of the mRNFL and mGCIPL in early relapsing–remitting multiple sclerosis (RRMS) (mean disease duration = 23 months), while [29] identifying GCIPL (GCL+IPL) atrophy as an early feature of MS, predominantly affecting the perifoveal zone. However, our conclusions do not fully coincide, likely due to differences in patient cohorts, instrumentation, and OCT acquisition protocols. Importantly, previous studies reached their conclusions through univariate statistical analysis of each variable, whereas our results are derived from overall data analysis performed by the classifier.

The comparison between AUC values (Figure 2) and SHAP-based feature ranking (Figure 5) also deserves comment. Although there is some overlap between the variables with the highest AUC values and those selected by the classifier (Figure 5), important differences also exist. These discrepancies may arise because AUC values reflect the relationship between controls and PwMS at the individual level. More detailed analysis of AUC values (Table 2) shows that:

- The median AUC value of the four analyzed structures, considering thickness measurements (mean, STD) across the six zones, ranged between 0.63 (IPL, mean variable) and 0.68 (IRL, mean variable).
- The median AUC value of the six anatomical zones, considering mean and STD for the four structures, ranged between 0.63 (Zone 5, Zone 6) and 0.69 (Zone 1).
- When analyzing mean and STD variables globally, the median AUC values were 0.66 and 0.65, respectively.

In conclusion, AUC values are useful to identify differences between controls and PwMS in individual variables; however, their average values across metrics (mean and STD) are quite similar. SHAP analysis is preferable, since the variables are analyzed jointly and selected recursively, ranking the input variables that maximize classifier accuracy. This highlights the value of explainable AI in extracting clinically meaningful insights beyond conventional univariate analysis.

Other studies using multicenter OCT datasets have also endorsed the value of this technique in diagnosing early-stage MS. For example, in a multicenter study with participants from 28 centers [28], it was demonstrated that significant thinning of the IRL was observed in individuals with MS (disease duration > 1 year) versus controls. In an international multicenter study spanning 11 sites, inter-eye differences of 5 μm in the RNFL and of 4 μm in the macular ganglion cells + IPL were found to be a very promising criteria for identifying unilateral optic nerve lesions [29]. In [28] the longitudinal stability of using inter-eye differences in the pRNFL and GCIPL as reliable markers of optic nerve lesions in PwMS is demonstrated. In [22], the authors used datasets from two medical centers, applying a dimensionality reduction method for feature selection combined with an SVM classifier. They identified the GCL and INL as the most influential layers; however, in one dataset, patient disease duration was not reported and, in the other, mean duration was 7.67 ± 1.37 years.

Cohort-wise evaluation showed stronger performance on the MSH dataset than on the RCH dataset. In particular, the model retained reasonable discrimination for control cases but showed reduced sensitivity for MS cases in the RCH cohort. This suggests that part of the performance degradation may be associated with inter-cohort variability, potentially including differences in acquisition protocol, image quality, population characteristics, or annotation distribution. These results highlight the importance of external validation and suggest that future work should focus on improving robustness through cohort harmonization, domain adaptation, or fine-tuning using additional data from under-represented acquisition settings.

Nevertheless, our study has limitations. The sample sizes from each center were not balanced, and there were differences in the disease durations between hospitals that may have influenced the results, and mismatched EDSS scores indicate clinical heterogeneity across centers. In most cases, both eyes of each subject have been considered, so the principle of independence of the statistical tests used is not strictly met. Although clinical histories were carefully reviewed, the possibility of subclinical ON episodes in the included eyes cannot be excluded. Moreover, our analysis was restricted to structural OCT data acquired with a single device and protocol.

Future studies should explore whether combining structural metrics (mean, STD, and inter-eye differences) with complementary modalities such as OCT angiography and visual evoked potentials could further enhance diagnostic accuracy. Additionally, if cohort size allows, it would be of interest to investigate the combined behavior of these three structural variables (mean, STD, and inter-eye difference) in AI-based diagnostics [30].

In conclusion, our results suggest that OCT, when analyzed using explainable AI methods, can support highly accurate MS diagnosis in a multicenter setting.

## Figures and Tables

**Table 1:** Study participant demographics and disease characteristics.

	PwMS			Controls		
	MSH	RCH	Statics	MSH	RCH	Statics
**N (eyes)**	98	14	-	135	58	-
**Male/female eyes**	21/77	2/12	*p* = 0.53	34/101	8/50	*p* = 0.078
**Age (years)**	42.54 ± 10.12	35.14 ± 9.43	0.019	48.20 ± 12.22	44.77 ± 11 .89	0.051
**Disease duration (years)**	1.42 ± 0.72	6.82 ± 2.57	<0.001	NA	NA	NA
**EDSS (median) [range]**	1.28 (0–3)	2.40 (1–6.5)	<0.001	NA	NA	NA

**PwMS:** people with multiple sclerosis. **MSH:** Miguel Servet Hospital. **RCH:** Ramón y Cajal Hospital. **EDSS:** Expanded Disability Status Scale. **NA:** not applicable.

**Table 2:** Detailed analysis of the AUC values.

	Variable: MEAN				Variable: STD				
	RNFL	GCL	IPL	IRL	RNFL	GCL	IPL	IRL	Median of Each Zone
Zone 1	0.73	0.74	0.72	0.72	0.73	0.66	0.67	0.53	0.72
Zone 2	0.69	0.72	0.69	0.69	0.72	0.52	0.62	0.61	0.69
Zone 3	0.68	0.63	0.58	0.68	0.64	0.67	0.65	0.70	0.66
Zone 4	0.68	0.62	0.58	0.69	0.65	0.68	0.63	0.69	0.67
Zone 5	0.63	0.65	0.59	0.65	0.61	0.65	0.61	0.61	0.62
Zone 6	0.58	0.63	0.59	0.62	0.61	0.68	0.68	0.67	0.63
Median of variable per layer	RNFL: 0.68	GCL: 0.64	IPL: 0.59	IRL: 0.69	RNFL: 0.65	GCL: 0.67	IPL: 0.64	IRL: 0.64	
Median of variable	MEDIAN: 0.67				STD: 0.65				
Median values per layer, considering variables MEAN and STD									
RNFL	0.66								
GCL	0.65								
**IPL**	**0.63**								
**IRL**	**0.68**								

**Table 3:** Performance of the 5 classifiers tested.

Method	Accuracy (%)	Sensitivity (%)	Specificity (%)
SVM-RBF	84.59	71.43	92.23
SVM-Linear	79.34	52.68	94.82
LR	77.38	52.68	91.71
LDA	78.03	50.89	93.78
KNN	81.97	64.29	92.23

## Data Availability

Data are available upon reasonable request to the corresponding author, due to ethical reasons.
